# Space-Time Analysis to Identify Areas at Risk of Mortality from Cardiovascular Disease

**DOI:** 10.1155/2015/841645

**Published:** 2015-10-04

**Authors:** Poliany C. O. Rodrigues, Emerson S. Santos, Eliane Ignotti, Sandra S. Hacon

**Affiliations:** ^1^Public Health and Environment Program, National School of Public Health (ENSP), FIOCRUZ, 21041-210 Rio de Janeiro, RJ, Brazil; ^2^Faculty of Medical Sciences, University of the State of Mato Grosso (UNEMAT), 78200-000 Cáceres, MT, Brazil; ^3^Department of Geography, Federal University of Mato Grosso (UFMT), 78060-900 Cuiabá, MT, Brazil

## Abstract

This study aimed at identifying areas that were at risk of mortality due to cardiovascular disease in residents aged 45 years or older of the cities of Cuiabá and Várzea Grande between 2009 and 2011. We conducted an ecological study of mortality rates related to cardiovascular disease. Mortality rates were calculated for each census tract by the Local Empirical Bayes estimator. High- and low-risk clusters were identified by retrospective space-time scans for each year using the Poisson probability model. We defined the year and month as the temporal analysis unit and the census tracts as the spatial analysis units adjusted by age and sex. The Mann-Whitney *U* test was used to compare the socioeconomic and environmental variables by risk classification. High-risk clusters showed higher income ratios than low-risk clusters, as did temperature range and atmospheric particulate matter. Low-risk clusters showed higher humidity than high-risk clusters. The Eastern region of Várzea Grande and the central region of Cuiabá were identified as areas at risk of mortality due to cardiovascular disease in individuals aged 45 years or older. High mortality risk was associated with socioeconomic and environmental factors. More high-risk clusters were observed at the end of the dry season.

## 1. Introduction

About 17 million cardiovascular disease (CVD) related deaths occur annually worldwide [1]. In Brazil, pathologies related to CVD are among the leading causes of death in individuals older than 45 years [2]. In Mato Grosso, heart disease is the second leading cause of death, and its prevalence has shown a trend for an increase, particularly among the elderly [3, 4]. In the cities of Cuiabá and Várzea Grande, CVD has been the major cause of death in the past 5 years, accounting for about 1,000 deaths per year [2].

Middle-aged individuals and the elderly are population at risk for CVD because many CVD risk factors are more prevalent during these stages of life. Moreover, increasing age is associated with a gradual decrease of the physiological resilience of the human body [5].

Several epidemiological studies have shown that almost 80% of patients with heart disease have classic risk factors. These include hypertension, dyslipidemia, obesity, diabetes, advanced age, male sex, family history, and certain lifestyle-related behaviors [6, 7]. Socioeconomic vulnerability, quality of and access to health services, and exposure to heat waves, high temperatures, and air pollution can also be important risk factors for CVD [8–11].

The relationships between the various factors related to the development of a disease can be observed through the distribution of these factors in the geographic space. Mapping health problems and identifying populations at risk of certain diseases and risk factors can be useful management action tools for planning, monitoring, and surveillance in public health [12].

The designation of priorities based on the space-time distribution of a disease enables better resource allocation, implementation of prevention strategies, or even emergency treatment for some diseases [13]. This study aimed to identify areas with a high and low risk of mortality from CVD among middle-aged and elderly individuals living in the urban areas of Cuiabá and Várzea Grande between 2009 and 2011.

## 2. Methods

### 2.1. Study Design

This is an ecological spatiotemporal study of CVD mortality rates within clusters that were identified by a risk classification methodology.

### 2.2. Population and Study Area

The study population included individuals aged 45 years and older living in the urban areas of Cuiabá and Várzea Grande who died from CVD (as classified by Chapter IX of the 10th Revision of the International Classification of Diseases-ICD-10-codes I00 to I99) between 2009 and 2011.

**Figure 1:**
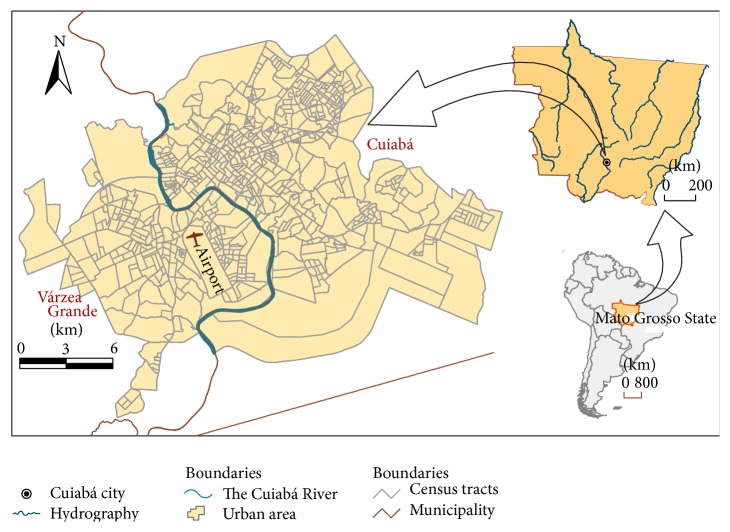
Study area: the urban areas of Cuiabá and Várzea Grande.

Cuiabá and Várzea Grande (Figure 1) are the most important cities within the metropolitan area of the State of Mato Grosso. These cities, separated physically by the Cuiabá River, form a conurbation with a population of about 820,000 inhabitants, corresponding to 90% of the total population of the metropolitan area. Approximately 98% of the population is concentrated within the urban areas. Furthermore, inhabitants aged 45 years and older represent approximately 60% of the total population of the 2 cities. The last census reported an increase in the rate of aging and life expectancy of this population [14].

### 2.3. Data Source

Mortality data were obtained from the Mortality Information System of the Brazil Unified Health System (SIM/SUS), given by the Health Department of the State of Mato Grosso. Information on the population, socioeconomics data* shapefiles* of the municipalities, and an aggregate of census tracts of 2010 census data were derived from the Brazilian Institute of Geography and Statistics.

Temperature and humidity records were obtained from the National Institute of Meteorology (INMET) [15], and aerosol optical depth (AOD) data, through which the concentrations of fine particulate matter (PM_2.5_) were estimated, were derived from the Aerosol Robotic Network (AERONET) [16].

### 2.4. Variables

We selected income ratio, education, and availability of basic services as proxy indicators of social and economic status of the residents.

The income ratio variable indicates situations of inequality in the census tracts. It was obtained by dividing the proportion of individuals with an income below the minimum wage by the proportion of individuals with an income 5 times above the minimum wage. A higher income ratio indicates a bad economic situation.

The education variable indicates the educational level of the population within the census tracts. It was obtained by assessing the ratio of the numbers of literate individuals aged 15 years or older in the census tract over the total population of the census tract, multiplied by 100. A high value for this variable indicates a higher educational level.

The “availability of basic services” variable refers to the availability of regular garbage collection and sanitation services within the neighborhood. This variable was obtained by dividing the number of households within “open sewer” and/or accumulated garbage in the surroundings by the total number of households in the census tract, multiplied by 100. A high value for basic services indicates poor sanitation conditions.

Temperature range, humidity, and PM_2.5_ were selected as environmental variables as they have well-known associations with CVD mortality.

The temperature range variable indicates variations in temperatures. It was obtained by subtracting the minimum from the maximum temperature. The humidity variable refers to the proportion of humidity in the environment during a certain period. PM_2.5_ is a mixture of liquid and solid particles suspended in the air derived from combustion [17]. It was estimated by converting the values of AOD (500 nm) using the equation *y* = 5 + (AOD × 40) [18].

### 2.5. Ethical Considerations

The Ethics Committee of the National School of Public Health (CAAE 18634613.0.0000.5240) approved this study.

### 2.6. Data Analysis

We analyzed deaths caused by CVD among individuals aged 45 years or older residing in the urban areas of the cities of Cuiabá and Várzea Grande from 2009 to 2011, which corresponded to 92% of the entire population including rural area. We geocoded the individuals' home addresses and grouped them by census tract for further analysis.

**Figure 2:**
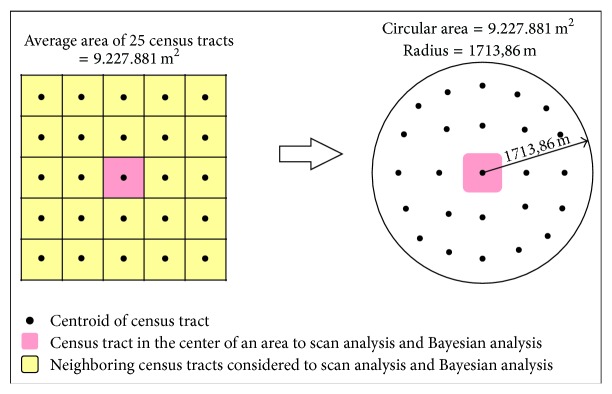
Radius setting scheme for Bayesian analysis and scan statistics.

We calculated mortality rates for each census tract from 2009 to 2011 to characterize the spatial distribution of CVD related deaths in the study area. The Local Empirical Bayes estimator using the weighted mean of the crude mortality rate within a location and the mortality rate of neighbors within each sector was performed to analyze these rates. This offered significant stability for the mortality rates, taking into consideration the random fluctuations in data derived from small areas [19]. The neighborhood matrix for the Bayesian estimation contained all neighbors within a distance of 1,713.86 meters to the centroid of each census tract. This distance is equal to the radius of a circle with an area equal to the average of 25 census tracts (9,227,871 m^2^) or 2 neighbors on each side of each census tract (Figure 2).

To identify the spatiotemporal clusters for a high and low risk of death due to CVD, statistical tests of spatial scans were performed using SaTScan 9.3 software (http://www.satscan.org/). This technique depends on the likelihood ratio between areas. It performs the spatial scan by moving a cylindrical window at the centroid of each census tract, where the base is the circular geographical area around the centroid and the cylinder height is time. Inside each cylinder, the observed and expected number of CVD deaths by the chosen probability model is calculated, resulting in the RR for each area [20]. Thus, it is possible to identify spatiotemporal clusters using a value that represents how an area is more or less susceptible to having the presence of the event (e.g., deaths related to CVD) when compared to the other studied areas.

The variables included in the space-time scan analysis were population data, number of cases (adjusted for age and sex), and the Cartesian coordinates (UTM Projection-Zone 21 South, metric units) of the census tract centroids. We performed a retrospective analysis for each year, using a Poisson probability model with a circular radius of 1,713.86 meters (Figure 2) and considering a cluster with up to 50% of the population at risk. The time unit of the analysis was the year and month of the occurrence, and the formation of clusters was limited to a minimum of 1 and a maximum of 4 months annually. Monte Carlo simulations were replicated 999 times to create confidence intervals and envelopes.

After the space-time scan, we created a database containing only the census tracts that were within a cluster of high or low risk in 2 or 3 of the analyzed years. We grouped these census tracts into 2 categories, those belonging to a low-risk cluster and those belonging to a high-risk cluster. A nonparametric Mann-Whitney *U* test was used to compare the means of the socioeconomic and environmental variables between the different risk categories using the SPSS 20.0 software.

The socioeconomic variables were only analyzed spatially because available data represented the average of the 3 years (2009–2011) for the census tracts. In contrast, the environmental variables were only analyzed temporally, since the available data represented the monthly averages of the entire study area. The values assumed for the environmental variables were the averages of data that correlated with each cluster's year and month of occurrence.

## 3. Results

Between 2009 and 2011, 2,762 deaths from CVD in individuals aged 45 years or older and residing in the urban areas of the cities of Cuiabá and Várzea Grande were observed. The distributions of deaths by age group were 13%, 21%, 27%, and 35% among those aged 45–54 years, 55–64 years, 65–74 years, and ≥75 years, respectively. Ischemic heart disease and cerebrovascular disease were the main causes of death, accounting for 25% and 32% of CVD deaths, respectively.

**Figure 3:**
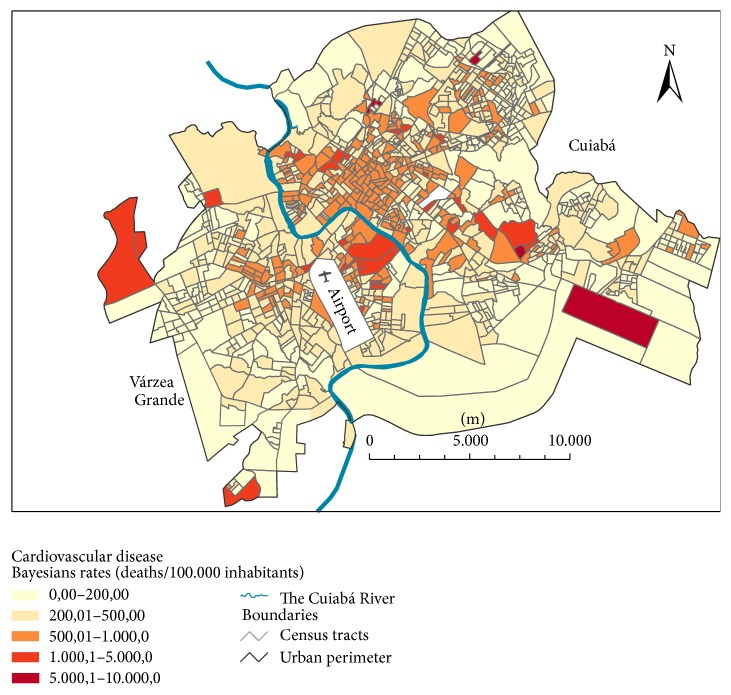
Spatial distribution of CVD mortality rates in adults aged 45 years or older.

The average mortality rate obtained by the Local Empirical Bayes estimator was 370 deaths per 100,000 inhabitants for the 2 cities from 2009 to 2011. We observed high CVD mortality areas (1,000–10,000 deaths per 100,000 inhabitants) in the Eastern and Western regions of Várzea Grande and in the Southeastern and Midwestern regions of Cuiabá. In both municipalities, we found areas with intermediate rates of CVD mortality (500–1,000 deaths per 100,000 inhabitants) in the central regions (Figure 3).

The Cuiabá and Várzea Grande conurbation contained 1166 census tracts within its urban area; of these, 62.7% (731 census tracts) had a relative risk (RR) for CVD mortality of <1 and 37.3% (435 census tracts) had an RR > 1. The average RR was 0.99 (standard deviation 1.6), and 25% of the census tracts had RRs > 1.4.

**Figure 4:**
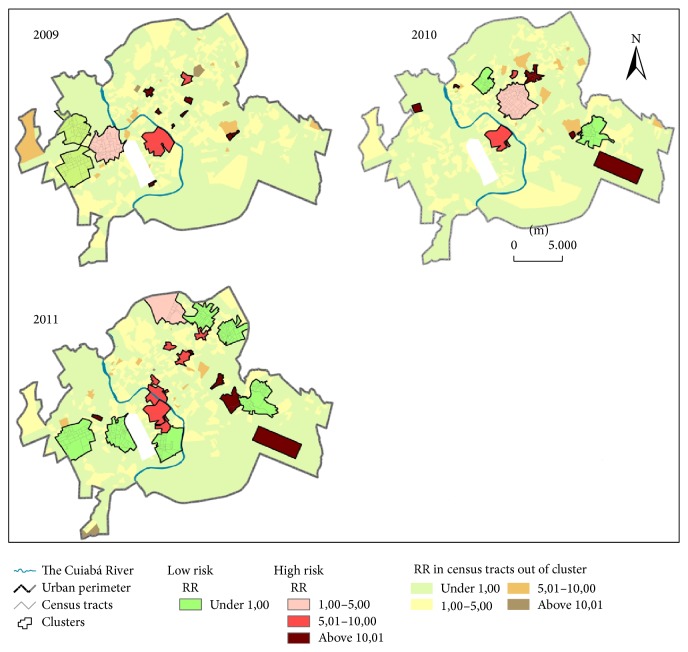
Spatial distribution of high and low relative risk (RR) clusters of CVD mortality.

A total of 13, 10, and 20 high-risk clusters and low-risk clusters were detected in 2009, 2010, and 2011, respectively. In 2009, we identified 11 high-risk clusters encompassing 81 census tracts and 2 low-risk clusters encompassing 66 census tracts. In 2010, 8 high-risk clusters encompassing 101 census tracts and 2 low-risk clusters encompassing 47 census tracts were identified. Finally, in 2011, we identified 14 high-risk clusters encompassing 67 census tracts and 6 low-risk clusters encompassing 211 census tracts. High-risk areas for CVD mortality were observed in central and Eastern Várzea Grande and in central and Southeastern Cuiabá (Figure 4).

During the entire study period, only 78 census tracts (distributed in 23 clusters) were within the high-risk clusters or low-risk clusters for at least 2 to 3 years. A total of 19 clusters encompassing 50 census tracts were classified as high risk and 4 clusters encompassing 28 census tracts as low risk.

About 79% of high-risk clusters were detected during the dry season (between June and November), while 75% of low-risk clusters were observed in the rainy season (February to May).

High-risk clusters were found in areas where the mean income ratios were higher and where higher average temperatures and higher levels of fine particulate matter (PM_2.5_) were observed. In contrast, low-risk clusters were areas where the mean humidity levels were high (Table 1).

## 4. Discussion

The areas at risk of CVD mortality in Cuiabá and Várzea Grande differed based on the socioeconomic factors of their residents and environmental factors. Barcellos and Sabroza [13] attribute the existence of diseases concentration areas primarily to local living conditions. This hypothesis assumes that the geographical space is associated with factors related to the development of diseases and its distribution among various social groups.

We found high-risk clusters for CVD mortality during periods with a higher temperature range (12 to 13°C), lower humidity (68 to 70%), and higher concentrations of PM_2.5_ (16 to 19 *μ*g/m^3^). Climate variability influences human health directly or indirectly [17]. Local environmental conditions related to temperature, humidity, air pollutants, and land use can accentuate the weakness of the body in fighting diseases by increasing inflammation and creating favorable conditions for the development of a disease [21, 22].

Rapid temperature changes and low humidity are associated with disease exacerbation and CVD mortality [17, 23]. This can be explained by the physiological exhaustion caused by prolonged exposure to thermal stress over many consecutive days [24]. Cheng and Su [25] and Huang et al. [24] argue that there is a strong relationship between thermoregulation and the circulatory regulation of an individual. This means that, in environmental situations with normal or moderate changes, the human organism can adapt more easily; however, when abrupt and intense changes occur, there is an overload, which affects the individual's resilience, eventually leading to illness and sometimes even death.

In the cities of Cuiabá and Várzea Grande, the effects of climatic events overlap with the intensification of fires. During the dry season (from June to November), several localities suffer from increased air pollution caused by wildfires and the burning of household waste in backyards and vacant lots [26]. Furthermore, the geographical location within a depression, combined with the lack of rain, reduced wind speed, and temperatures during this period make the region a target of recurrent episodes of heat exchange. In this process, a layer of cold air is retained in the region near the earth's surface, reducing the local temperature and hindering the dispersion of pollutants. This results in the formation of a haze consisting of many pollutants (smog) from industrial emissions, cars, and wildfires [27].

PM_2.5_ has high toxicity. Fine particles (smaller than 2.5 *μ*m) can reach the deeper parts of the respiratory system and even cross the epithelial barrier, triggering an inflammatory response [28, 29]. Several studies have noted increased morbidity and mortality from CVD related to this pollutant. Evidence suggests that short-term PM_2.5_ exposure increases the risk of arterial thrombosis, including myocardial infarction and stroke, while chronic exposure increases the formation of atherosclerotic plaques, reducing life expectancy by a few years [30]. In Brazil, an ecological study in the Brazilian Amazon found an association between PM_2.5_, described as the percentage of hours of exposure to PM_2.5_ with a concentration of >25 mg/m^3^, and CVD mortality in the elderly (*β* adjusted = 0.05; *p* = 0.002) [4].

Census tracts with higher income ratios had high-risk clusters for CVD mortality in our study. There are similar results in other cities of Brazil. Some authors, in Porto Alegre [31], in Pernambuco [32], and in Rio de Janeiro [33–36], observed higher CVD mortality risks associated with socioeconomic inequalities and precarious sanitation services.

Income inequality generates a geographic concentration of poverty that is associated with many social disadvantages (e.g., low education level, low income, and precarious sanitation services) and increases the exposure of residents to various diseases [37, 38]. According to Fiscella and Tancredi [39], certain lifestyle-related behaviors (e.g., smoking cessation, diet improvement, and physical activity) are not very prevalent in low socioeconomic status; that is why the population can be more vulnerable to diseases. Silva and Ribeiro [40] add that the health risks related to extreme weather conditions also increase in these areas, both because of the houses built with materials and techniques that hinder thermal insulation and because of financial constraints that affect the ability of residents to obtain suitable devices for regulating unfavorable microclimate conditions (e.g., air conditioning, fans, and heaters).

The area with the greatest risk of CVD mortality was located in the central area of the Eastern region of Várzea Grande. This region consists of several suburban neighborhoods. It has a high population density and various social problems related to water supply, public transport, access to health services, and violence, while having a residential population with unequal economic profiles [41].

*Limitations*. The main limitation of this study was the use of secondary data and, therefore, the accuracy and validity of the variables that were used. These limitations might have affected our results by underestimating or overestimating associations; however, information from secondary databases has been widely used in epidemiological studies worldwide [42].

The reliability of mortality data in the Midwest region of Brazil is satisfactory, since only an average of 4% of cases had a poorly defined cause of death from 2009 to 2011 [2]. In the cities of Cuiabá and Várzea Grande, the proportion of deaths with ill-defined causes followed the decreasing national trend, suggesting an improvement in mortality information in this region. In Cuiabá and Várzea Grande city, the proportions of deaths with poorly defined causes during the study period were on average 1.4% and 0.9%, respectively [2]. Data from the Brazilian Institute of Geography and Statistics and National Institute of Meteorology have been described as reliable by several Brazilian studies [43, 44].

Diseases, especially chronic diseases, reflect a summation of individual and biological factors that manifest themselves for different reasons and times throughout an individual's life. It is important to note that this study took into account a limited number of indicators and that there are other CVD risk factors such as hypercholesterolemia, hypertension, smoking, alcohol consumption, physical inactivity, poor diet, and obesity, which were not accounted for [45]. Various studies have shown that these factors tend to occur simultaneously [46–48], increasing the risk of CVD, especially when associated with poor living conditions, variations in environmental parameters, and exposure to air pollution [49].

## 5. Conclusions

In conclusion, individuals aged 45 years or older from the Eastern region of Várzea Grande and the Cuiabá central region had the highest risk of CVD mortality. Socioeconomic conditions and environmental characteristics were strongly associated with this increased risk. Moreover, more high-risk clusters were observed during the end of the dry season and the beginning of the rainy season (August to November).

A geographic element's incorporation in this epidemiological study enabled the identification of areas at risk, which enables the redirection of epidemiological surveillance and environmental health measures.

We therefore suggest that it would be beneficial to establish policies for economic development and social empowerment that value health promotion and education, primarily the adoption of healthy habits as well as the monitoring of indicators related to air pollution and other individual risk factors related to lifestyle.

## Figures and Tables

**Table 1:** Comparison of socioeconomic and environmental factors by risk categories, Cuiabá and Várzea Grande (2009–2011).

	Mean	Confidence interval		Mann-Whitney *U* test	
		Lower limit	Upper limit	Value	*p* value
Socioeconomics variables					
Income ratio (%)					
Low risk	0.03	0.02	0.05	5.10	0.03
High risk	0.13	0.01	0.26		
Total	0.07	0.02	0.11		
Availability of basic services (%)					
Low risk	12.38	5.45	19.30	0.01	0.93
High risk	11.86	3.77	19.94		
Total	12.20	6.99	17.40		
Education (%)					
Low risk	70.69	67.37	74.01	0.32	0.57
High risk	68.67	60.95	76.39		
Total	69.96	66.57	73.36		
Environmental variables					
Temperature range (°C)					
Low risk	10.03	9.64	10.41	60.44	<0.001
High risk	12.49	11.98	13.01		
Total	10.91	10.51	11.32		
Humidity (%)					
Low risk	73.58	72.49	74.67	26.64	<0.001
High risk	69.10	67.79	70.41		
Total	71.97	71.01	72.93		
PM_2.5_ (*μ*g/m^3^)					
Low risk	14.24	12.79	15.69	7.67	0.01
High risk	17.39	15.75	19.03		
Total	15.37	14.24	16.50		
